# MAT2A-Mediated S-Adenosylmethionine Level in CD4^+^ T Cells Regulates HIV-1 Latent Infection

**DOI:** 10.3389/fimmu.2021.745784

**Published:** 2021-09-20

**Authors:** Xiaofan Yang, Ting Huang, Tiantian Wang, Hongbo Gao, Haitao Zhang, Wen Peng, Jiacong Zhao, Shujing Hu, Panpan Lu, Zhongsi Hong, Bo Li, Kai Deng

**Affiliations:** ^1^Institute of Human Virology, Key Laboratory of Tropical Disease Control of Ministry of Education, Zhongshan School of Medicine, Sun Yat-sen University, Guangzhou, China; ^2^Department of Immunology, Zhongshan School of Medicine, Sun Yat-sen University, Guangzhou, China; ^3^School of Medicine, Sun Yat-sen University, Shenzhen, China; ^4^Department of Infectious Diseases, Fifth Affiliated Hospital, Sun Yat-sen University, Zhuhai, China; ^5^Department of Biochemistry, Zhongshan School of Medicine, Sun Yat-sen University, Guangzhou, China

**Keywords:** CD4^+^ T cell, MAT2A, HIV-1, latent infection, one-carbon flux

## Abstract

Antiretroviral drugs effectively halt HIV-1 replication and disease progression, however, due to the presence of a stable viral latent reservoir, the infection cannot be cured by antiretroviral drugs alone. Elucidating the molecular mechanisms underlying HIV-1 latent infection remains a critical hurdle that precludes the development of novel therapeutic strategies aiming for a potential functional cure. Cellular metabolism has been reported to affect HIV-1 replication in CD4^+^ T cells, but it remains largely unclear whether it is involved in the regulation of HIV-1 latency. Here, we performed a sub-pooled CRISPR library knockout screen targeting 1773 metabolic-related genes in a cell model of HIV-1 latent infection and found that Methionine Adenosyltransferase 2A (MAT2A) contributes to HIV-1 latency. MAT2A knockout enhanced the reactivation of latent HIV-1 while MAT2A overexpression did the opposite. Mechanistically, MAT2A modulates HIV-1 latency through S-Adenosylmethionine (SAM)-mediated one-carbon flux. MAT2A knockout resulted in a significant downregulation of DNA and histone methylation at the HIV-1 5’-LTR. Importantly, we found that the plasma level of SAM is positively correlated with HIV-1 DNA in PBMCs from ART-treated infected individuals, suggesting SAM could serve as a potential biomarker for the latent viral reservoir. Overall, this study reveals an important role of MAT2A-mediated one-carbon metabolism in regulating HIV-1 latency and provides a promising target for the development of new strategies for a functional cure of HIV-1.

## Introduction

Due to the stable integration and silent transcription of the HIV-1 genome in the long-lived quiescent memory T lymphocytes, the host immune system fails to recognize and eliminate these small portion of HIV-1 infected cells, which are commonly referred as viral latent reservoirs (1–3). Although antiretroviral therapy (ART) can effectively suppress HIV-1 replication, it cannot eradicate the viral latent reservoirs and infected individuals have to remain on ART for life (4). Once the therapy is stopped, a viral rebound is inevitable in each case (4). Consequently, the viral latent reservoirs represent the major obstacle to HIV-1 eradication (5). The ‘shock and kill’ approach, which is based on the use of the latency reversal agents (LRAs) to reactivate the viral transcription and subsequent elimination of the virus *via* HIV-1 specific immune response is generally considered to be a promising strategy to eliminate the latent reservoirs (6). However, several LRAs have not achieved the expected results in clinical trials (7). The ‘block and lock’ approach, which aims to permanently silence all proviruses is another emerging strategy for HIV-1 functional cure (8, 9). Nevertheless, these strategies have not yet been clinically applied primarily because our understanding of the mechanisms underlying HIV-1 latent infection is still insufficient.

Several molecular mechanisms have so far been reported to contribute to the silencing of HIV-1 transcription in latently infected cells, including absence of essential transcriptional factors, transcriptional interference, heterochromatin or epigenetic alterations (10–14). As a typical retrovirus, the life cycle of HIV-1 is totally dependent on the host cell that it infects. Cellular metabolism has been shown to significantly affect HIV-1 replication in CD4^+^ T cells (15), however, it remains largely unclear whether it plays a role in the regulation of HIV-1 latency. Numerous studies have demonstrated that cellular metabolisms are involved in the activation, proliferation, differentiation, and immune response of CD4^+^ T lymphocytes (16, 17). The metabolic state of CD4^+^ T lymphocytes is accompanied by changes in metabolites which provide the necessary energy for the life cycle of HIV-1, such as viral replication and silencing. A recent study has shown that HIV-1 tends to infect metabolic-active CD4^+^ T cells independent of their activation phenotype and the susceptibility of CD4^+^ T cell subsets to HIV-1 matches their metabolic activity (18). HIV-1 infection promotes glucose uptake and glycolysis that in turn enhance HIV-1 infection and replication (19). Similarly, tryptophan metabolism promotes HIV-1 infection and transcription by the activation of a ligand-activated transcription factor aryl hydrocarbon receptor (AHR) in CD4^+^ T cells (20). There is also enough evidence to suggest that intracellular dNTP levels are critical for HIV-1 reverse transcription. A typical example is SAMHD1, which acts as a host restriction factor *via* reducing the dNTP levels in the cytoplasm to restrict HIV-1 infection in resting CD4^+^ T cells (21, 22). Taken together, cellular metabolism significantly affects the replication events of HIV-1 in CD4^+^ T cells. However, the roles of host cellular metabolism in the establishment and maintenance of HIV-1 latency remains poorly understood.

CRISPR (Clustered Regularly Interspaced Short Palindromic Repeats) screening is a powerful tool to identify genes contributing to cellular phenotypes and has been universally used to identify host restriction factors regulating host-virus interaction (23–26). In this study, we performed a sub-pooled CRISPR library knockout screen of 1773 metabolic-related genes in a HIV-1 latently infected cell line (J-Lat 9.2) and identified methionine adenosyltransferase 2A (MAT2A) as a novel host factor contributing to HIV-1 latency. We further showed that MAT2A deletion enhances the reactivation of HIV-1 latency by the inhibition of HIV-1 promoter LTR DNA and histone methylation through the deprivation of S-adenosylmethionine (SAM). Our results suggest that MAT2A plays an important role in the maintenance of HIV-1 latency and might be a novel target for the development of novel strategies against HIV-1.

## Materials & Methods

### Study Approval

Peripheral blood from healthy adult participants was collected by the Institutional Review Board of Guangzhou Blood Center (Guangzhou, Guangdong, China). Peripheral blood from HIV-1 infected patients on antiretroviral therapy was collected by The Fifth Affiliated Hospital of Sun Yat-sen University (Zhuhai, Guangdong, China). Approval of the research was signed by the Ethics Review Board of Sun Yat-Sen University, the Institutional Review Board of Guangzhou Blood Center, The Fifth Affiliated Hospital of Sun Yat-sen University. All human participants provided written informed consent for their participation in the study and agreed with the publication of the scientific results.

### Cell Culture

Jurkat cell line and J-Lat cell lines (J-Lat A2, J-Lat 6.3, J-Lat 8.4, J-Lat 9.2, J-Lat 10.6, J-Lat 15.4) were cultured in RPMI 1640 (Gibco) supplemented with 1% penicillin-streptomycin and 10% fetal bovine serum (FBS) (Gibco). The human embryonic kidney cell line HEK293T (CVCL_0063; ATCC) and TZM-bl cells (8129) were cultured in DMEM (Gibco) supplemented with 1% penicillin-streptomycin and 10% fetal bovine serum (FBS) (Gibco). All cells had been tested and confirmed to be mycoplasma-free by PCR assays. All cells were maintained in a clean incubator at 37°C and 5% CO2.

### CRISPR Screening

The human CRISPR metabolic gene knockout library (MeGKO library), which contains 4 sgRNAs per gene for 1773 human metabolic genes and 50 sgRNA targeted no gene as random control, was constructed using the lentiCRISPRV2 vector by Bo Li’s laboratory. The CRISPR screening was carried out according to Feng Zhang’s previously published protocol (27). Briefly, MeGKO library was used to package lentiviruses in HEK293T cells and then infected J-Lat 9.2 cells at a low multiplicity of infection (MOI< 0.3). After the selection of puromycin for 7 days, cells were treated with TNF-α (20 ng/mL) for 48 hours and then proceeded to sort GFP positive cells and GFP negative cells by FACS. Both GFP positive cells and GFP negative cells were harvested for extraction of genomic DNAs by using TIANamp Genomic DNA Kit DP304 (TIANGEN BIOTECH, BEIJING). PCRs were performed to amplify the total sgRNA sequences by using NEBNext High Fidelity PCR Master Mix, 2× (New England BioLabs, cat. no. M0541L) with the following primers: FP: ACGATACAAGGCTGTTAGAG; RP: CAAGTTGATAACGGACTAGC. The pooled PCR amplicons of sgRNA sequences were then processed for sequencing with HiSeq X Ten (Illumina) at Novogene (Beijing, China). The sgRNA reads were further analyzed by MAGeCK to obtain the enrichment of sgRNA as described previously (28, 29).

### CRISPR–Cas9-Mediated Gene Knockout

Knockout cells were generated by lentivirus-mediated CRISPR/Cas9 technology, according to Feng Zhang’s protocol (27). Briefly, single guided RNA (sgRNA) sequences were designed by using an online CRISPR design tool (http://www.e-crisp.org/E-CRISP/designcrispr.html). Individual sgRNAs were cloned into the lentiviral expression vectors, LentiCRISPR-V2 (addgene 52961) (27). Then the sgRNA-encoding CRISPR lentiviruses were prepared in HEK293T cells. J-Lat cell lines were infected with a single sgRNA-encoding CRISPR lentivirus individually and selected with puromycin (1 μg/ml, Sigma-Aldrich) for 4-7 days. The knockout efficiency was confirmed by western blot analysis. SgRNA sequences used in this study are provided in **Supplementary Table 1**. SgRNA targeting dummyguide (sgNT: 5’-ACGGAGGCTAAGCGTCGCAA-3’) was set as negative control (30).

### shRNA

ShRNA-expression vector (miR302d+backbone+stuffer+-thy-shRNA), which contains the puromycin resistance gene and red fluorescent protein (RFP), was a gift from Dr. Penghui Zhou’s laboratory (SYSU). ShRNA-target sequences were subcloned to shRNA-expression vectors and subjected to packaged lentiviruses in HEK293T cells and infect the indicated cells with the selection of puromycin. RT-PCR and western blotting were used to confirm the MAT2A expression. ShRNA-target sequences used in this study were as follows:

shLacZ: ATTCCGCCGATACTGACGGGCTC; shMAT2A-1: CTCAAGTTACTGTGCAGTATATG; shMAT2A-2: AGCAGTTGTGCCTGCGAAATACC.

### Flow Cytometry Analysis

The reactivation efficiency in J-Lat cells or BCL-2 cells was measured by the GFP-positive percentage by flow cytometry analysis. Single-cell suspensions were prepared and detected on a FACS LSRII (BD Biosciences) and data were analyzed by using the FlowJo software.

### Western Blot Analysis

One % SDS Hot Lysates Preparation was performed to lyse the cells, according to the instructions provided by Abcam. Briefly, after washed with cold phosphate-buffered saline (PBS), cells were lysed in hot 1% SDS Hot lysis buffer (10 mM Tris-HCl (pH8.0), 1%SDS, 1.0 mM Na-Orthovanadate, ddH2O) in 100 °C for 10 ~20 min. Then an ultrasonic cell disruptor was used to break all cell clusters until the lysate becomes clear (Ultrasound time 3 s, 10 s interval, ultrasonic 5 ~15 times, ultrasonic power: 40 kW). Finally, cell lysates were centrifuged for 5~10 minutes at 15000 ~17000 g and discard the cell pellet. The lysates were denatured in NuPAGE running buffer (Thermo Fisher) at 100°C for 10 min. Equal amounts of lysates were subjected to a 12.5% continuous SDS-PAGE to separate the proteins, transferred the protein to PVDF membranes (Merck Millipore), and probed with specific antibodies followed by secondary antibodies conjugated with horseradish peroxidase (HRP). ChemiDoc Touch (BIO-RAD) was used to detect the HRP signal with incubation of Electrochemiluminescence (ECL) (BIO-RAD). ImageLab (BIO-RAD) was used to analyze the blots.

### Real-Time PCR

Total RNA was extracted by using TRIzol reagent (ThermoFisher) and subjected to cDNA synthesis with HiScript III RT SuperMix for qPCR (+gDNA wiper) (Vazyme, Nanjing, China), according to the manufacturer’s instructions. The mRNA levels of MAT2A were performed with ChamQ Universal SYBR qPCR Master Mix (Vazyme, Nanjing, China) on an ABI QuantStudio5 (Thermo Fish) machine by using the following program: (i) 95°C 30 s, 1 cycle; (ii) 95°C 10 s; 60°C 15 s; 40 cycles; (iii) 72°C 5 min, 1 cycle. The relative expression of each gene was calculated by using the ΔΔCT method. The sequences of the primers were as follows:

MAT2A-qPCR-FP: ATGAACGGACAGCTCAACGG; MAT2A-qPCR-RP: CCAGCAAGAAGGATCATTCCAG.

### Ectopic Gene Expression

For ectopic gene expression, pCDH-CMV-MCS-EF1-Puro was used to construct the MAT2A-overexpression lentivirus vector. Briefly, CDS (coding sequence) of MAT2A was obtained from mRNA of Jurkat cells by reverse PCR reaction by using the following primers: MAT2A-Infusion-FP: CATAGAAGATTCTAGATGAACGGACAGCTCAACG; MAT2A-Infusion-RP: ATTTAAATTCGAATTTCAATATTTAAGCTTTTTGGGCAC. Then the DNA fragment was cloned into the pCDH-CMV-MCS-EF1-Puro vector by using an HB-infusion (TM) kit according to the manufacturer’s instructions (HANBIO, China). The MAT2A-encoding vector was used to packaged lentiviruses and infect the indicated cells with the selection of puromycin. The pCDH-CMV-MCS-EF1-Puro vector was used as a control. RT-PCR and western blotting were used to confirm the MAT2A expression.

### Measurement of SAM

For measurement of intracellular SAM, 10 million cells per sample were washed twice with cold 1×PBS and once more with cold 0.9% (w/v) NaCl. Cell pellets were collected by centrifuging at 500 g for 8 min at 4°C and suspended with 1 mL of pre-chilled methanol/acetonitrile/water solution (5:3:2, vol/vol/vol) and incubated at -80°C for 20 min. Then the lysates were centrifuged at 14,000 g for 20 min at 4°C and the SAM containing supernatants were transferred to a new tube and processed to measure the level of intracellular SAM by LC-MS analysis. For measurement of plasma SAM, 100 μL plasma each sample was added with 300 μL pre-chilled acetonitrile/water solution (4:1, v/v). Six concentrations of SAM standards [40 ppb, 200 ppb, 400 ppb, 800 ppb, 2000 ppb, 4000ppb (ppb=ng/ml)] were prepared with 50% acetonitrile/water solution (1:1, v/v). Another six of 90 μL plasma from each sample were taken and added 10 μL of 6 concentrations of the standards respectively and then added 300 μL of prechilled acetonitrile/water (4:1, v/v). plasm samples were vortexed for 5 minutes and centrifuged at 12000g for 10 minutes at 4°C and pellets were discarded. 200 μL of the supernatant in each sample was processed to measure the level of SAM by LC-MS analysis. The whole process is operated on ice or under 4°C. Optima-grade methanol, Optima-grade acetonitrile, S-(5′-Adenosyl)-L-methionine chloride dihydrochloride (A7007) were purchased from Sigma-Aldrich.

For SAM quantification, samples were analyzed at the Metabolomics Facility, Core Facilities of Medical Science, Zhongshan School of Medicine, Sun Yat-Sen University, China. Samples were analyzed by using QExactive (Thermo Fisher, CA).

### Generation of Latently HIV-1–Infected BCL2–Transduced Cells

BCL-2 transduced latent CD4+ T cells were generated as described previously (31). Briefly, primary CD4+T cells were isolated from the PBMCs of healthy donors with a human CD4+ T lymphocyte enrichment set (BD, USA) according to the manufacturer’s instructions. After co-stimulated with anti-CD3 and anti-CD28 antibodies (BioLegend), CD4+ T cells were transduced with BCL-2 and cultured in the cytokine-free medium for 4 weeks to obtain BCL-2-transduced cells. Then BCL-2–transduced cells were activated and expanded in the presence of exogenous IL-2. Subsequently, BCL-2-transduced cells were activated again and infected with a reporter virus NL4-3-Δ6-drEGFP (CM6). Following cultured in the cytokine-free medium for 1 month, cells were sorted to obtain GFP-negative cells by cell sorting (FACSAria II; Becton Dickinson). The expression of GFP in GFP-negative cells was used to assess the reversal of latency by flow cytometry analysis.

### siRNA Transfection and Luciferase Reporter Assays

TZM-BL cells were transfected with siRNA targeting MAT2A (siMAT2A: GTGGCAAAATCCCTTGTTA) by using Lipofectamine RNAiMAX (ThermoFisher) according to the manufacturer’s instruction. Random siRNA (siNC) was used as a control. After 48 hours, cells were stimulated with or without TNF-α (10 ng/mL) for 24 hours. Then the cells were lysed and measured with luciferase-reporter assay (Promega) by using a multiwell plate luminometer with an auto-injector (Promega) and analyzed by GloMax 96 Microplate Luminometer Software (Promega), according to the manufacturer’s instruction. Fold changes were calculated for each gene compared with siNC according to the light units.

### Methylation Analysis of HIV-1 5’-LTR

Genomic DNA was extracted from J-Lat 9.2 cells with MAT2A knockout or not by using the Tissue DNA Kit D3396 (Omega bio-tek). The extracted genomic DNAs were treated with bisulfite by using EZ DNA Methylation-Gold Kit D5005 (ZYMO RESEARCH), according to the manufacturer’s instructions. Bisulfite-treated DNA was amplified in PCR reactions by using Champagne Taq DNA Polymerase P122-d1 (Vazyme, Nanjing, China) with the following reaction conditions: 3 min denaturation at 95°C, 35 cycles of 30 s at 95°C, 15 s at 55°C, and 5 min at 72°C. The PCR products were cloned into a pMD™19-T vector (TAKARA) and sequenced to determine the methylation of CpG islands. Bisulfite-specific PCR primers for HIV-1 5’-LTR as follow:

Forward Primer (FP): AATAAAGGAGAGAATATTAGTTTGT; Reverse Primer (RP): CTCAAAACAAACTTTATTAAAACTTAAAC.

### Chromatin Immunoprecipitation (ChIP) Assays

ChIP assays were performed by using SimpleChIP^®^ Enzymatic Chromatin IP Kit (Magnetic Beads) (#9003, CST) according to the manufacturer’s instruction. Briefly, 4 x 10^6^ cells were prepared for each immunoprecipitation (IP) assay. MAT2A knockout J-Lat 9.2 cells and control cells were cross-linked with 1% formaldehyde and followed by quenching with 125 mM glycine. After washing with cold 1× PBS, cells were lysed with Buffer A supplemented with DTT (dithiothreitol) and Protease inhibitor cocktail (PIC) for 10 min on ice. After centrifugation, nuclear pellets were suspended with Buffer B supplemented with DTT and digested with 0.5 μL micrococcal nuclease per IP at 37°C for 20 min. After digestion, the suspension of nuclear pellet was suspended with ChIP buffer and processed to sonicate for 1 min (20 s on, 30 s off) at 30% amplitude by using SONICS VCX130 (sonicman, USA). One-tenth of the lysates per sample was proceeded to analyze the concentration and fragment length of the sheared chromatin DNA. For each IP preparation, the lysate contained 10 μg chromatin DNA was incubated with specific antibodies (anti-H3K9me3 antibody (Abcam, ab8898), anti-H3K4me3 antibody (Abcam, ab8580), anti-H3K27me3 antibody (Abcam, ab6002)) or control rabbit IgG at 4°C overnight. Besides, 2% volume of lysate each IP was used as the input sample to calculate the enrichment efficiency. 30 μL of ChIP-Grade Protein G Magnetic Beads was added to each IP sample at 4°C for 2 hours. IP samples were then washed with low-salt washing buffer three times and high-salt washing buffer for one time. The eluted samples were then reversed cross-linking with 200 mM NaCl and Proteinase K and processed to extract DNA. The input and immunoprecipitated DNA were further quantified by RT-PCR. The primers targeting HIV-1 5’-LTR nuc-1 in J-Lat 9.2 cells were showed as following: FP: CTGGGAGCTCTCTGGCTAAC; RP: AGACGGGCACACACTACTTG.

### Measurement of HIV-1 Proviral DNA

Genomic DNA was extracted and purified from PBMC with the Tissue DNA Kit D3396 (Omega bio-tek), according to the manufacturer’s instructions, in a final volume of 100 μ l. Real-time PCR was performed to measure HIV-1 proviral DNA by using the following primers and probe, described previously (32):

FP: ACATCAAGCAGCCATGCAAAT; RP: TCTGGCCTGGTGCAATAGG, Probe: VIC-CTATCCCATTCTGCAGCTTCCTCATTGATG-TAMRA.

### Measurement of HIV-1 mRNA

HIV-1 RNA was extracted by using TRIzol reagent (ThermoFisher) and subjected to cDNA synthesis with HiScript III RT SuperMix for qPCR (+gDNA wiper) (Vazyme, Nanjing, China), according to the manufacturer’s instructions. Real-time PCR was performed by using AceQ^®^ Universal U+ Probe Master Mix V2 (Vazyme, Nanjing, China) on an ABI QuantStudio5 (Thermo Fish) machine using the following program: (i) 37°C 2 min, 1 cycle; (ii) 95°C 5 min, 1 cycl2; (iii) 95°C 10 s; 60°C 30 s; 40 cycles; (iii) 72°C 5 min, 1 cycle. HIV-1 mRNAs were detected by using the following primers and probe, modified from Shan et al. (33): FP: CAGATGCTGCATATAAGCAGCTG (9501 to 9523); RP: TTTTTTTTTTTTTTTTTTTTTTTTGAAGCAC [9629 to the poly(A) tail]; probe: 6-carboxyfluorescein (FAM)-CCTGTACTGGGTCTCTCTGG-MGB (9531 to 9550). A standard curve was generated for each PCR reaction by using a TOPO plasmid as described in Shan et al.’ article (33).

### Statistical Analysis

Data for independent experiments are representative of 2–5 independent experiments with similar results and presented as mean ± SD unless otherwise stated. Student’s t-test (two-tailed unpaired) was performed to determine statistical significance by using GraphPad Prism. A P-value of ≤ 0.05 was considered to be statistically significant and represented as an asterisk (*).

## Results

### A Sub-Pooled CRISPR Screen Identifies Metabolic Genes Controlling HIV-1 Latency

**Figure 1:**
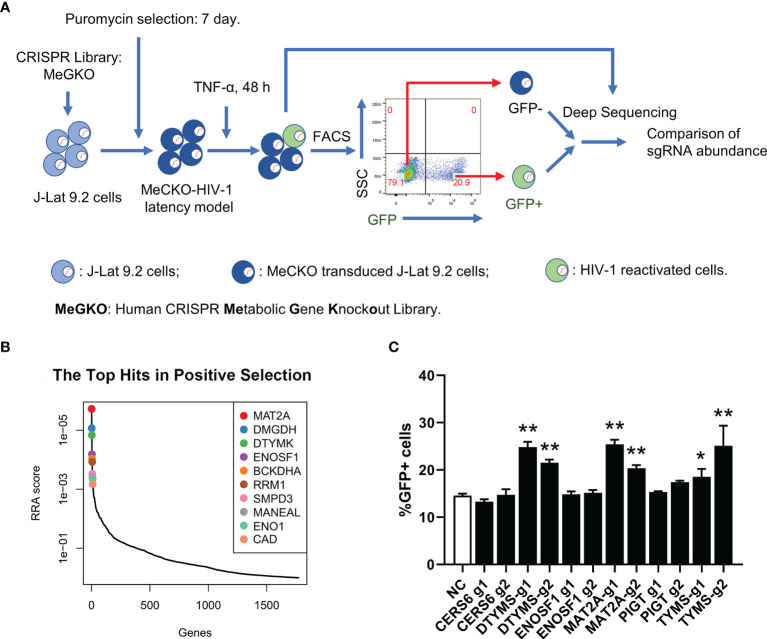
A sub-pooled CRISPR screening of metabolic genes. **(A)** Schematic overview of CRISPR/Cas9 screen for the identification of metabolic genes controlling HIV-1 latency. **(B)** Distribution of RRA scores in GFP+ cells *versus* GFP- cells with positive selection. Top 10 hits were showed. **(C)** Flow cytometry analysis of the percentage of GFP positive cells in J-Lat 9.2 cells with TNF-α stimulation for 24 h after knockout of the candidate genes. Data were presented as mean ± SD from three independent experiments (Student’s t-test, *p < 0.05, **p < 0.01).

To identify potential metabolic pathways contributing to HIV-1 latency, we performed a sub-pooled CRISPR library knockout screen in a cell line model of HIV-1 latent infection, J-Lat 9.2, which was derived from Jurkat cells and harboring a single integrated proviral genome with a green fluorescent protein (GFP) reporter (34). In J-Lat 9.2 cells, the HIV-1 provirus was latent under basal conditions but could be reactivated upon LRAs (latency-reversing agents) stimulation usually using PMA or TNF-α targeting NF-κB signaling and quantified by the level of induced GFP expression. The human CRISPR metabolic gene knockout library (MeGKO library) contains 4 sgRNAs per gene for 1773 human metabolic genes and 50 non-targeting sgRNAs as random controls. The schematic overview of our CRISPR screening is shown in **Figure 1A**. Deep sequencing data of sgRNA counts were analyzed by MAGeCK (28, 29). The top 10 hits of GFP+ cells versus GFP- cells with the positive selection which indicated sgRNAs enriched in GFP+ cells are listed in **Figure 1B**. To further validate the results of our initial screening, several candidate genes were selected and knocked out in J-Lat 9.2 using the lentiviral-vectored CRISPR/Cas9 method. We found that the knockout of methionine adenosyltransferase 2A (MAT2A), a cellular enzyme that catalyzes the formation of S-Adenosylmethionine (SAM) from methionine and ATP, led to a significant reactivation of latent HIV-1 by TNF-α (**Figure 1C**). Therefore, MAT2A was selected for further investigation.

### MAT2A Regulates HIV-1 Reactivation in Latently Infected Cells

**Figure 2:**
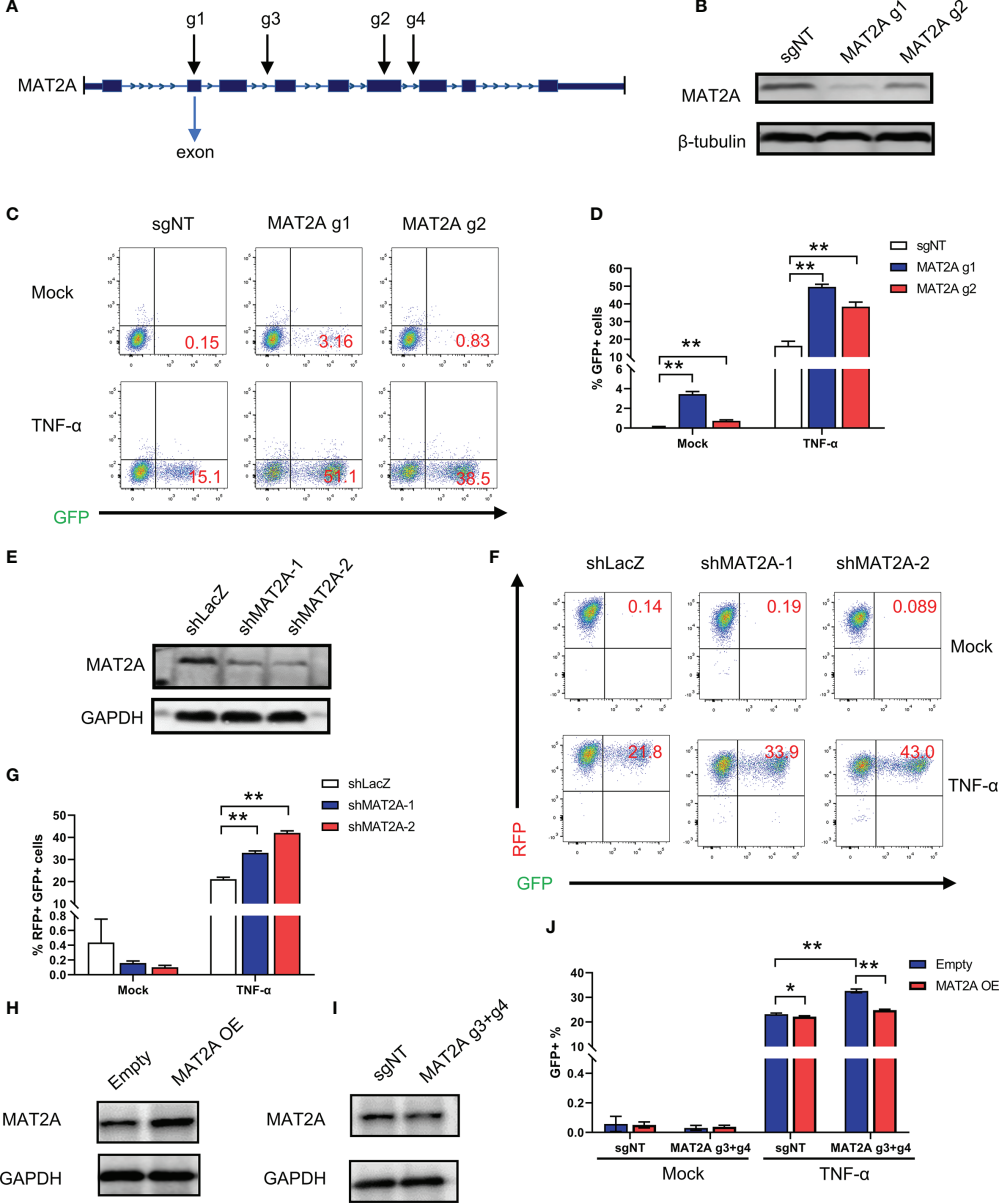
MAT2A regulates HIV-1 reactivation in latently-infected cells. **(A)** Schematic diagram of sgRNAs targeting the MAT2A gene. **(B)** Western blot analysis of the knockout efficiency of MAT2A in J-Lat 9.2 cells by MAT2A g1 and MAT2A g2 knockout, respectively. sgNT knockout cells served as control. **(C, D)** The MAT2A knockout J-Lat 9.2 cells were stimulated with or without TNF-α for 24 h GFP-positive cells were measured by flow cytometry. **(E)** Western blot analysis of the knockdown efficiency of MAT2A in J-Lat 9.2 cells by shMAT2A-1 and shMAT2A-2 knockdown, respectively. shLacZ knockdown cells served as control. **(F, G)** The MAT2A knockdown J-Lat 9.2 cells were stimulated with or without TNF-α for 24 h GFP-positive cells were measured by flow cytometry. **(H, I)** Western blot analysis of the expression of MAT2A in J-Lat 9.2 cells with MAT2A overexpression (MAT2A OE) or MAT2A g3 + g4 knockout. **(J)** MAT2A knockout in J-Lat 9.2 cells by MAT2A g3 + g4 and then followed by the overexpression of MAT2A. GFP-positive cells were measured by flow cytometry. Data were presented as mean ± SD from three independent experiments (Student’s t-test, *p < 0.05, **p < 0.01).

To further confirm whether MAT2A is involved in the regulation of HIV-1 latency, we designed two sgRNAs (MAT2A g1 and MAT2A g2) targeting two different exons in the MAT2A gene to knock out MAT2A in J-Lat 9.2 cells (**Figure 2A**). As was shown in **Figure 2B**, the level of MAT2A protein was sharply decreased after knockout, as MAT2A g1, which targets the first exon, showed a higher knockout efficiency. MAT2A knockout resulted in significant upregulation of latency reversal with or without TNF-α stimulation as compared to the non-targeting sgRNA (sgNT) (**Figures 2C, D**). The effect of MAT2A g1 was higher than MAT2A g2, which was consistent with their knockout efficiency.

Given that off-target effects are a major drawback of the CRISPR/Cas9 system (35), we verified MAT2A’s effect in J-Lat 9.2 cells by knockdown experiments with shRNA lentiviruses. Western blot showed that the MAT2A protein was efficiently knocked down by shMAT2A-1 and shMAT2A-2 as compared to the control shRNA (shLacZ) (**Figure 2E**). As expected, MAT2A knockdown enhanced latent HIV-1 reactivation comparing to shLacZ (**Figures 2F, G**). However, under basal condition, we didn’t observe latent HIV-1 reactivation by FACS analysis after MAT2A knockdown (**Figures 2F, G**), likely due to the residual effect of MAT2A with the shRNA system.

Additionally, we performed a rescue experiment to further validate the loss of function mutation result of MAT2A. To avoid MAT2A-specific sgRNA targeting the exons of MAT2A overexpression construct, we designed two sgRNA (MAT2A g3, MAT2A g4) targeting two different introns in MAT2A. We first transfected MAT2A g3 and g4 (MAT2A g3+g4) into J-Lat 9.2 cells to obtain a long-range region deletion of MAT2A and then overexpressed MAT2A. Compared with the sharply knockout efficiency of MAT2A g1 and g2 targeting the exons, MAT2A g3 + g4 showed only a slight reduction in the expression level of the MAT2A protein (**Figure 2I**). Nevertheless, after MAT2A g3+g4 knockout, latent HIV-1 was significantly reactivated (**Figure 2J**). After MAT2A overexpression, the level of MAT2A protein was increased (**Figure 2H**), which subsequently led to a resistance to HIV-1 latency reversal by TNF-α (**Figure 2J**). Moreover, overexpressed MAT2A in MAT2A g3+g4 knockout cells rescued the phenotype caused by MAT2A g3+g4 (**Figure 2J**). Taken together, these collective results confirmed that MAT2A plays an important role in the maintenance of HIV-1 latency.

In mammalian cells, there are two other MAT-encoding genes, including MAT1A and MAT2B. MAT1A is mainly located in adult quiescent hepatocytes whereas MAT2A and MAT2B are widely distributed in extrahepatic tissues (36, 37) (**Supplementary Figure 1**). To investigate whether MAT1A and MAT2B regulate HIV-1 latency, we knocked out MAT1A and MAT2B individually in J-Lat 8.4 and J-Lat 9.2 cells. sgNT was served as the negative control while MAT2A g1 and g2 as the positive control. After knockout, there was no significant effect on the reactivation of latent HIV-1 among MAT1A, MAT2B, and sgNT in J-Lat 8.4 and J-Lat 9.2 cells with or without PMA stimulation (**Supplementary Figure 1**). Therefore, these results indicated that MAT1A and MAT2B play no significant role in latency reversal of J-Lat 8.4 and J-Lat 9.2 cells.

**Figure 3:**
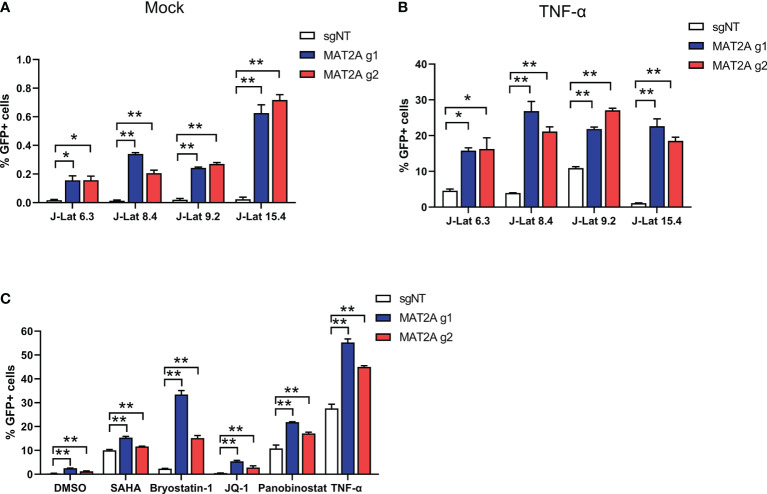
MAT2A deletion promoted latent HIV-1 reactivation. **(A, B)** The effect of MAT2A on HIV-1 latency reactivation is independent of the proviral integration site. Flow cytometry analysis of GFP positive cells in MAT2A knockout J-Lat cell lines with or without TNF-α stimulation. sgNT knockout cells served as control. **(C)** MAT2A knockout significantly facilitated LRA-mediated latent HIV-1 reactivation in J-Lat 9.2 cells. MAT2A knockout J-Lat 9.2 cells were treated with DMSO, SAHA, Bryostatin-1, JQ1, Panobinostat and TNF-α for 24 h GFP-positive cells were measured by flow cytometry. Data were presented as mean ± SD from three independent experiments (Student’s t-test, *p < 0.05, **p < 0.01).

We also tested the effect of MAT2A knockout in other J-Lat cell lines (J-Lat 6.3, 8.4 and 15.4) that harbored different HIV-1 insertion sites. The results were consistent with J-Lat 9.2 cells (**Figures 3A, B**), indicating that the effect of MAT2A knockout on latency reversal is independent of proviral integration sites. It has been reported that a single LRA failed to effectively reactivate latent HIV-1 *in vivo* and hence the suggestion that a combination of LRAs may be a promising strategy. Therefore, we assessed HIV-1 reactivation upon MAT2A-depletion with different LRAs acting through distinct mechanisms in J-Lat 9.2 cells. The results showed MAT2A deletion effectively enhanced the reactivation of latent HIV-1 with stimulation of all of LRAs we used (**Figure 3C**), indicating MAT2A can be a novel therapeutic target for developing promising LRAs.

### MAT2A Regulates HIV-1 Latency Through SAM-Mediated One-Carbon Flux

**Figure 4:**
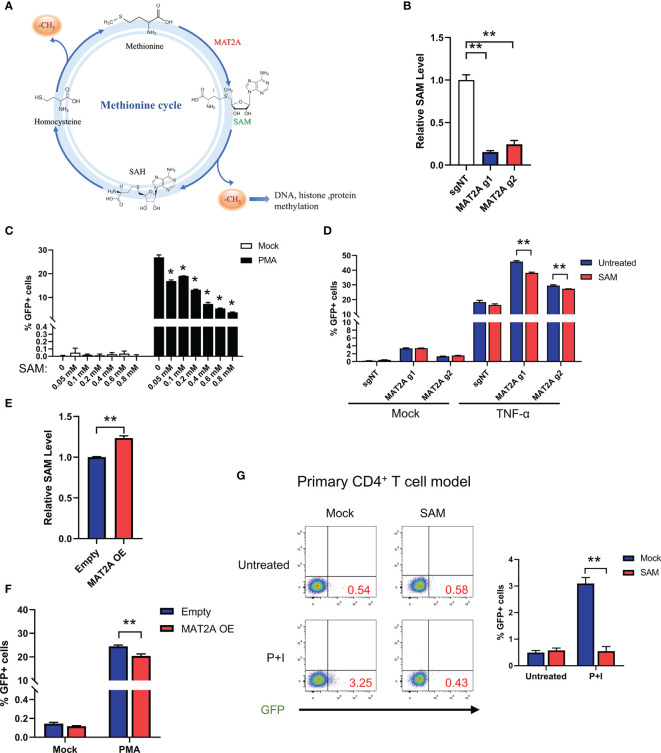
MAT2A regulates HIV-1 latency through SAM-Mediated One-Carbon Flux. **(A)** Overview of the methionine cycles. **(B)** LC-MS analysis of intracellular SAM in J-Lat 9.2 cells with or without MAT2A knockout. **(C)** J-Lat 9.2 cells were treated with different doses of SAM and then stimulated with or without PMA. GFP-positive cells were measured by flow cytometry. **(D)** J-Lat 9.2 cells with or without MAT2A knockout were treated with SAM for 2 days and stimulated with PMA for 24 h GFP-positive cells were measured by flow cytometry. **(E)** LC-MS analysis of intracellular SAM of J-Lat 9.2 cells with MAT2A overexpression. **(F)** Flow cytometry analysis of GFP positive cells in MAT2A overexpressed J-Lat cell lines with or without PMA stimulation. **(G)** BCL-2 cell model treated with or without SAM (0.8 mM) for 2 days and stimulated with PMA and ionomycin for 24 h Reactivation efficacy of latent HIV-1 was assessed by measured GFP by flow cytometry. Data were presented as mean ± SD from three independent experiments (Student’s t-test, *p < 0.05, **p < 0.01).

Next, we investigated the mechanism underlying MAT2A’s role in HIV-1 latency. Traditionally, the role of MAT2A is to catalyze the formation of SAM(S-Adenosylmethionine), which is a critical player in one-carbon flux and functions as the major methyl donor for cellular methylation reactions (**Figure 4A**). Therefore, we hypothesized that MAT2A regulates HIV-1 latency by modulating intracellular SAM generation. We indeed found that MAT2A knockout resulted in a dramatic decrease in intracellular SAM levels as measured by LC-MS (**Figure 4B**). When exogenous SAM was added in J-Lat 9.2 cells, the increasing doses of SAM strongly suppressed latency reversal (**Figure 4C**), suggesting the importance of SAM in the regulation of HIV-1 latency. Furthermore, addition of exogenous SAM in MAT2A knockout J-Lat 9.2 cells sufficiently rescued the phenotype induced by MAT2A knockout (**Figure 4D**). Additionally, overexpression of MAT2A increased SAM production and suppressed the reactivation of latent HIV-1 (**Figures 4E, F**). These results suggested that SAM suppressed latency reversal in J-Lat cells. To further validate these results in a primary T cell model of HIV-1 latency, we generated BCL-2-transduced primary CD4^+^ T cells and established latency by GFP-encoded pseudovirus infection (31). After SAM treatment, latent HIV-1 exhibited significant resistance to reactivation by PMA and ionomycin(**Figure 4G**), confirming that MAT2A maintains HIV-1 latency through modulating the availability of intracellular SAM.

Because SAM is derived from L-methionine (Met) (**Figure 4A**), we explored whether Met is also involved in regulating HIV-1 latency. J-Lat 9.2 cells were cultured in a medium containing low, normal, and high concentrations of Met according to the previous study (38). However, different concentrations of Met did not influence latency reversal after PMA stimulation (**Supplementary Figure 2**), suggesting that the effect of methionine cycle on HIV-1 latency is largely dependent on MAT2A but not Met.

### MAT2A Regulates HIV-1 Latency by Modulating DNA and Histone Methylation at Proviral 5’-LTR

**Figure 5:**
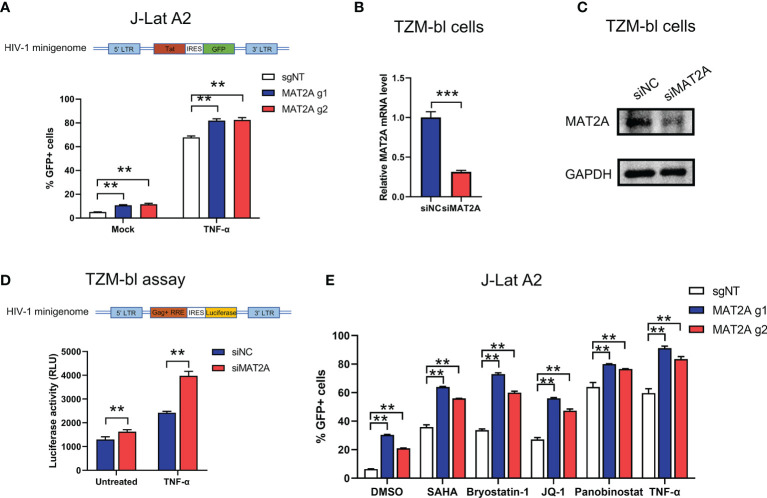
MAT2A regulates latent HIV-1 reactivation by modulating promoter activity. **(A)** Flow cytometry analysis of GFP-positive cells in MAT2A knockout J-Lat A2 cells with or without TNF-α stimulation. sgNT knockout cells served as control. **(B, C)** The knockdown efficiency by MAT2A siRNA was confirmed by RT-qPCR and Western blot. **(D)** TZM-bl cell line was transfected with siNC or siMAT2A for 48 h, then stimulated with or without TNF-α for 24 h Cells were harvested and the activity of luciferase was measured. **(E)** MAT2A knockout J-Lat A2 cells were treated with DMSO, SAHA, Bryostatin-1, JQ1, Panobinostat and TNF-α for 24 h GFP-positive cells were measured by flow cytometry. Data were presented as mean ± SD from three independent experiments (Student’s t-test, **p < 0.01, ***p < 0.001).

HIV-1 5’-Long terminal repeats (5’-LTR) serves as the promoter of the integrated provirus, which tightly controls the transcription of HIV-1 genome by hijacking the host cellular transcriptional apparatus (39). Transcriptional silencing of LTR activity is a major mechanism contributing to the establishment and maintenance of the viral latent reservoir. Therefore, we sought to explore whether MAT2A regulates HIV-1 latency through direct modulation of HIV-1 promoter activity. MAT2A KO in J-Lat A2 cells, which contains a ‘LTR-Tat-IRES-GFP’ minigenome, resulted in upregulation of latency reversal with or without TNF-α stimulation (**Figure 5A**), suggesting MAT2A’s effect is independent of viral protein production. We then knocked down MAT2A by siRNA in the TZM-bl cell line which carries a β-galactosidase gene and a luciferase reporter gene under the control of the HIV-1 LTR promoter (**Figures 5B, C**) and measured promoter activity by luciferase assay. After MAT2A knockdown, HIV-1 promoter activity was enhanced with or without TNF-α stimulation as compared to the control (**Figure 5D**). Furthermore, we treated MAT2A knockout J-Lat A2 cell lines with different LRAs which act through distinct mechanisms on LTR to promote proviral transcription, respectively. We observe that MAT2A knockout significantly facilitated LRA-mediated latency reversal when combining with all LRAs tested (**Figure 5E**). This result indicated that MAT2A deletion enhanced the reactivation of latent HIV-1 through separate mechanisms different from commonly used LRAs. Collectively, these data indicated that MAT2A regulates HIV-1 latency *via* modulating HIV-1 5’-LTR activity.

**Figure 6:**
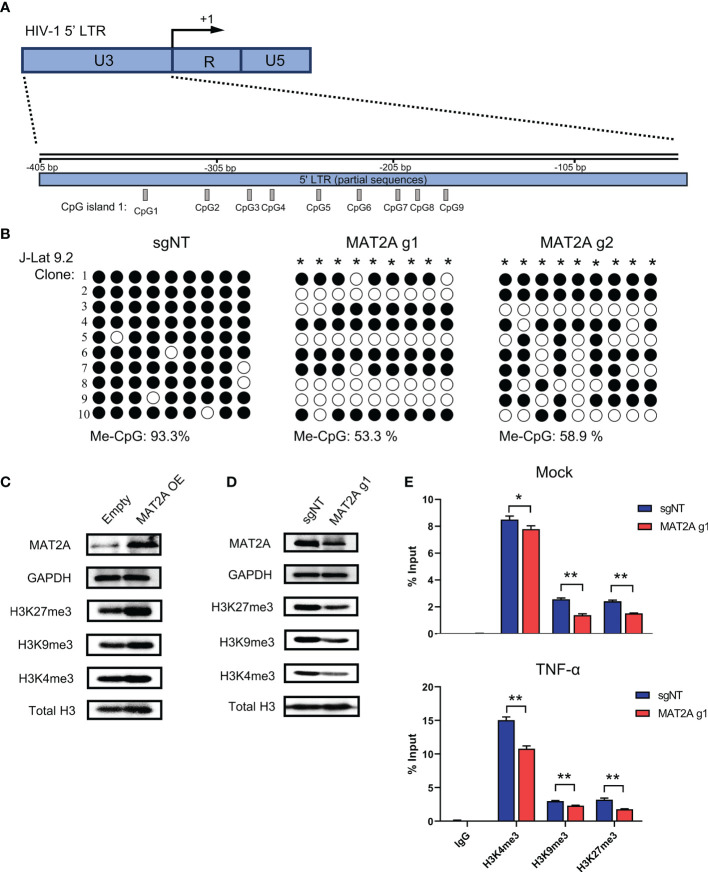
MAT2A regulates HIV-1 latency by modulating LTR DNA and histone methylation. **(A)** Schematic overview of the CpG island 1 at HIV-1 5’ LTR. **(B)** Bisulfite-mediated methylcytosine mapping of latent HIV-1 in J-Lat 9.2 cells with MAT2A knockout. sgNT knockout cells served as control. Each column represents one CpG position. Open circles represent nonmethylated CpG residues and closed circles represent methylated CpG residues. Asterisks indicate cytosines with statistically significant methylation. **(C, D)** Western blot analysis of MAT2A and methylated histones in J-Lat 9.2 cells with MAT2A overexpression **(C)** or knockout **(D)**. GAPDH and total histone H3 were used as loading controls. **(E)** Measurement of the levels of methylated histones at HIV-1 5’ LTR nucleosome nuc-1. ChIP-qPCR assays with antibodies against H3K4me3, H3K9me3, and H3K27me3 were performed in MAT2A knockout J-Lat 9.2 cells with or without TNF-α stimulation. Data were presented as mean ± SD from three independent experiments (Student’s t-test, *p < 0.05, **p < 0.01).

It has been well recognized that methylation modifications on proviral 5’-LTR DNA and histone are important mechanisms involved in HIV-1 latency. Given that SAM is the major methyl donor for cellular methylation reactions, we proposed that MAT2A regulates HIV-1 latency by modulating the availability of SAM, which subsequently affects the methylation profile of the 5’-LTR DNA and histone. To test whether MAT2A regulates CpG methylation, we knocked out MAT2A in J-Lat 9.2 cells and analyzed CpG island methylation of the 5’-LTR by bisulfite-mediated methylcytosine mapping (**Figure 6A**). MAT2A knockout resulted in a roughly 50% decrease in methylation as compared to the control (**Figure 6B**), demonstrating that modulating CpG island methylation is likely a cause of MAT2A’s role on HIV-1 latency. We overexpressed or knocked out MAT2A in J-Lat 9.2 cells and measured the levels of histone methylation (H3K4me3, H3K9me3, H3K27me3) by Western blot analysis. In J-Lat 9.2 cells, overexpression of MAT2A significantly elevated the levels of H3K4me3, H3K9me3, and H3K27me3 (**Figure 6C**), with H3K27me3 showing the most upregulation as compared to H3K4me3 and H3K9me3. On the contrary, with the knockout of MAT2A, the levels of H3K4me3, H3K9me3, and H3K27me3 were dramatically downregulated (**Figure 6D**). Proviral 5’-LTR contains two nucleosomes (nuc-0 and nuc-1), with nuc-1 having the closest proximity downstream of the transcription start site, therefore epigenetic modification of nuc-1 has the most critical impact on HIV-1 transcription. ChIP-qPCR showed that the levels of H3K4me3, H3K9me3 and H3K27me3 at nuc-1 of 5’-LTR were significantly reduced with or without TNF-α stimulation (**Figures 6E, F**), suggesting that MAT2A regulates HIV-1 latency through modulating the status of histone H3 methylation at HIV-1 promoter. Taken together, we can conclude that MAT2A regulates HIV-1 latency by reprogramming the methylation profile of HIV-1 LTR, both on the levels of DNA and histones.

### SAM Could Serve as a Biomarker for HIV-1 Latent Reservoir

**Figure 7:**
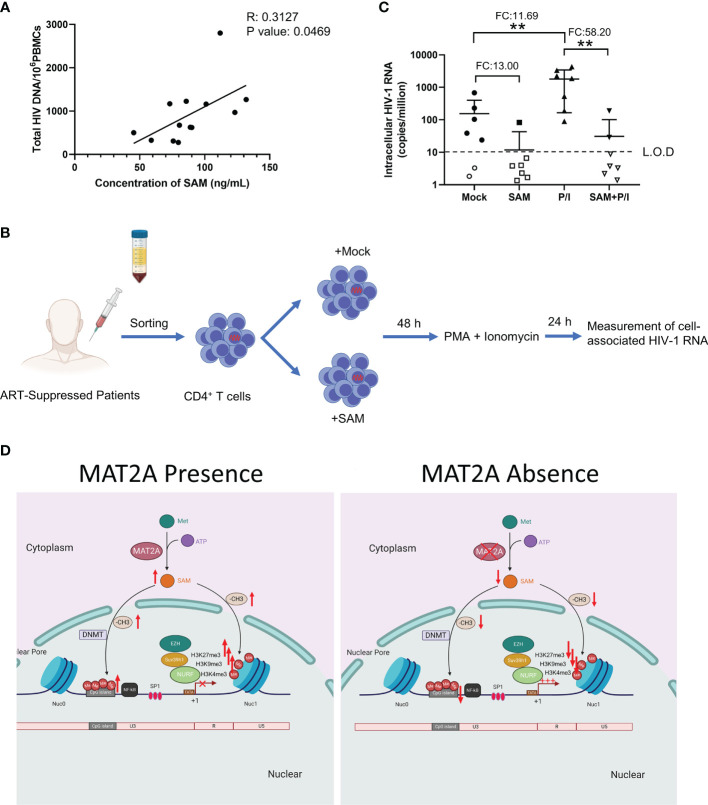
SAM plays an important role in HIV-1 latent infection in CD4^+^ T cells of ART-treated HIV-1 infected individuals *ex vivo*. **(A)** Correlation between the levels of serum SAM and HIV-1 proviral DNA in PBMC from ART-treated patients. The levels of serum SAM were analyzed by LC-MS. The levels of HIV-1 proviral DNA were detected by RT-PCR. **(B)** Experimental design of SAM’s inhibitory effect on HIV-1 transcription. **(C)** CD4^+^ T cells from ART-treated HIV-1-infected patient samples were treated with DMSO or SAM and then stimulated with or without PMA and ionomycin combination. Cell-associated HIV-1 RNA was measured by VQA. FC, fold change; L.O.D, low of detection. Student’s t-test, **p < 0.01. **(D)** Schematic diagram showing the role of MAT2A in the regulation of HIV-1 latency.

Finally, we sought to confirm whether SAM, which is catalyzed by MAT2A, is involved in HIV-1 latency in ART-treated infected individuals. To test this, we measured the levels of plasma SAM and total HIV-1 DNA in peripheral blood mononuclear cells (PBMCs) from 13 ART-treated patients, who had undetectable viral loads (<50 copies/ml) for at least 6 months. Plasma level of SAM was positively correlated with total HIV-1 DNA in PBMCs (R^2^: 0.3127, P-value: 0.0469) (**Figure 7A**), suggesting that SAM might facilitate the establishment of HIV-1 latent reservoir and serve as a potential biomarker. To investigate whether SAM could suppress latency reversal ex vivo, we purified CD4^+^ T cells from seven HIV-1-infected individuals on suppressive ART and treated the cells with exogenous SAM for 48 h (**Figure 7B**). After SAM treatment, cell-associated HIV-1 RNA was significantly reduced, either in the presence or absence of PMA and ionomycin (**Figure 7C**), suggesting that SAM directly inhibits latency reversal, which again highlights the important roles of MAT2A and its catalytic product SAM in the regulation of HIV-1 latency. Taken together, the above data indicate that MAT2A regulates HIV-1 latency through modulating the availability of SAM, which subsequently reprogram DNA and histone methylation profile at the proviral promoter (**Figure 7D**).

## Discussion

One of the obstacles that hinder clinical application of the “shock and kill” strategy is the low efficiency of LRA to reactivate latent HIV-1 *in vivo* (7). Therefore, combination of LRAs may be required to achieve sufficient latency reversal. For example, chaetocin, a specific inhibitor of Suv39H1, induces stronger latency reversal when combined with vorinostat or prostratin than using alone (40). Our results show that MAT2A knockout can effectively enhance latency reversal, and more importantly, it effectively cooperates with different classes of LRAs, suggesting that MAT2A inhibitors can be developed as a novel class of LRA that can also serve as a beneficial candidate for LRA combinations.

The biological function of MAT2A is to catalyze the generation of methyl donor SAM. Previous studies have shown that MAT2A deficiency could lead to a decrease in the level of intracellular SAM (41). In our study, we confirmed that MAT2A knockout led to a significant downregulation of intracellular SAM. More importantly, our results demonstrate the important role of SAM in HIV-1 latent infection. Despite the high efficacy of ART treatment, up to 50% of infected individuals suffer from mild forms of HIV-1-associated neurocognitive diseases (HAND) (42). One possible explanation is that the integrated proviruses in the central nervous system (CNS) (such as microglial cells, which are the main HIV-1 reservoir in the brain) continue to replicate because of anatomical sanctuaries (43). A recent report showed that SAM is a safe, effective treatment for depression among people living with HIV-1 (44). However, whether SAM plays a therapeutic role by suppressing the potential replication of HIV-1 in CNS remains to be further explored.

SAM is an essential and universal substrate for cellular methylation reaction. Several studies have shown that the alteration in intracellular SAM can affect DNA methylation and histone methylation modification (45, 46). For example, Liu and colleagues observed that hypoxia induces genomic DNA demethylation by reducing the steady-state SAM level both *in vitro* and *in vivo* (47). Wang et al. showed that MAT2A knockout caused changes in histone methylation modification (46). In accordance, our results showed that MAT2A knockout results in a significant decrease in the level of DNA and histone methylation at HIV-1 5’-LTR (**Figure 6**). Repressive methylation modifications at proviral promoters play a key role in the establishment and maintenance of HIV-1 latency (48, 49). Recently, Jiang et al. reported that compared to long-term ART-treated individuals, DNA sequence in close proximity to genome-intact proviral sequences from elite controllers showed a higher degree of cytosine methylation, indicating that DNA methylation can suppress proviral transcription *in vivo* for a long time and achieve a state of deep latency (50). Our findings demonstrated that MAT2A knockout significantly downregulates the methylation of CpG island at the HIV-1 promoter, suggesting that MAT2A is actively influencing the establishment and maintenance of HIV-1 latency. Several pieces of evidence have shown that the repressive histone methylation modification of the proviral promoter enforces HIV-1 latency. Lindqvist et al. showed that the heterochromatin marks H3K9me3 and H3K27me3 gradually became more prominent throughout the time-course of primary CD4^+^ T cells (51), consistent with HIV-1 gradually entering a latent state over time. Similarly, Nguyen et al. also indicated that both H3K27 and H3K9 methyltransferases play essential roles in the establishment and maintenance of HIV-1 latency in resting memory cells *ex vivo* and *in vivo (*52). Our data confirmed that MAT2A deletion results in a significant decrease in the levels of H3K9me3 and H3K27me3, while MAT2A overexpression does exactly the opposite. These results are consistent with a recent study that also demonstrated MAT2A deletion in different cancer cells, suggesting MAT2A globally regulates the histone methylated marks (46). Another recent study suggested that H3K4me3 does not directly activate the expression of the target genes but antagonizing DNA methylation and H3K27me3 to relieve the inhibition of these two modifications on the genes (53). In our report, we found that the repressive histone methylated marks and active histone methylated marks were both altered by MAT2A, suggesting the repressive methylated histone marks might play a leading role in the maintenance of HIV-1 latency. Overall, the metabolic control of SAM production by MAT2A in CD4^+^ T cells may serve as an upstream switch for the establishment or maintenance of HIV-1 latency *via* methylation-related modifications on proviral LTR. Pharmalogical inhibition of MAT2A activity *in vivo* could potentially alter the fate of latently infected cells, and represents a new direction for future HIV-1 cure road maps.

In summary, we identified a metabolic gene MAT2A as a novel host factor contributing to the maintenance of HIV-1 latency. This finding highlights the important role of one-carbon metabolism in the regulation of HIV-1 latency and may lead to the development of novel strategies for HIV-1 functional cure.

## Data Availability Statement

The original contributions presented in the study are included in the article/**Supplementary Material**. Further inquiries can be directed to the corresponding author.

## Ethics Statement

The studies involving human participants were reviewed and approved by the Ethics Review Board of Sun Yat-Sen University, the Institutional Review Board of Guangzhou Blood Center, The Fifth Affiliated Hospital of Sun Yat-sen University. The patients/participants provided their written informed consent to participate in this study.

## Author Contributions

KD and XY designed the study, interpreted the data, and wrote the manuscript. XY carried out the experiments and analyzed the data. TH, TW, HG, HZ, WP, JZ, PL, SH, and BL provided technical and intellectual assistance. KD supervised the study. All authors contributed to the article and approved the submitted version.

## Conflict of Interest

The authors declare that the research was conducted in the absence of any commercial or financial relationships that could be construed as a potential conflict of interest.

## Publisher’s Note

All claims expressed in this article are solely those of the authors and do not necessarily represent those of their affiliated organizations, or those of the publisher, the editors and the reviewers. Any product that may be evaluated in this article, or claim that may be made by its manufacturer, is not guaranteed or endorsed by the publisher.

## Supplementary Material

Supplementary Figure 1 MAT1A and MAT2B did not affect the reactivation of latent HIV-1 in J-Lat cells. **(A)** Schematic diagram of the distribution of MAT1A, MAT2A, and MAT2B *in vivo*. **(B, C)** Knockout of MAT1A, MAT2A, and MAT2B in J-Lat 8.4 and J-Lat 9.2 cells, respectively. Flow cytometry analysis of GFP positive cells in J-Lat 8.4 and J-Lat 9.2 cells after TNF-α stimulation. sgNT knockout cells served as control.

Supplementary Figure 2 Effect of methionine on the reactivation of HIV-1 latency in J-Lat 9.2 cells. J-Lat 9.2 cells were starved with methionine (Met) or supplemented with the indicated concentrations of Met for 7 days and then treated with or without TNF-α for 24 h. GFP-positive cells were measured by flow cytometry. Data were presented as mean ± SD from three independent experiments (Student’s t-test, *p < 0.05, **p < 0.01).

Supplementary Table 1 SgRNA sequences.
