# Supplementary figures and images for: MAT2A-Mediated S-Adenosylmethionine Level in CD4+ T Cells Regulates HIV-1 Latent Infection
Source: Front Immunol. 2021 Sep 20;12:745784. doi: 10.3389/fimmu.2021.745784 (PMC8488394; doi:10.3389/fimmu.2021.745784)

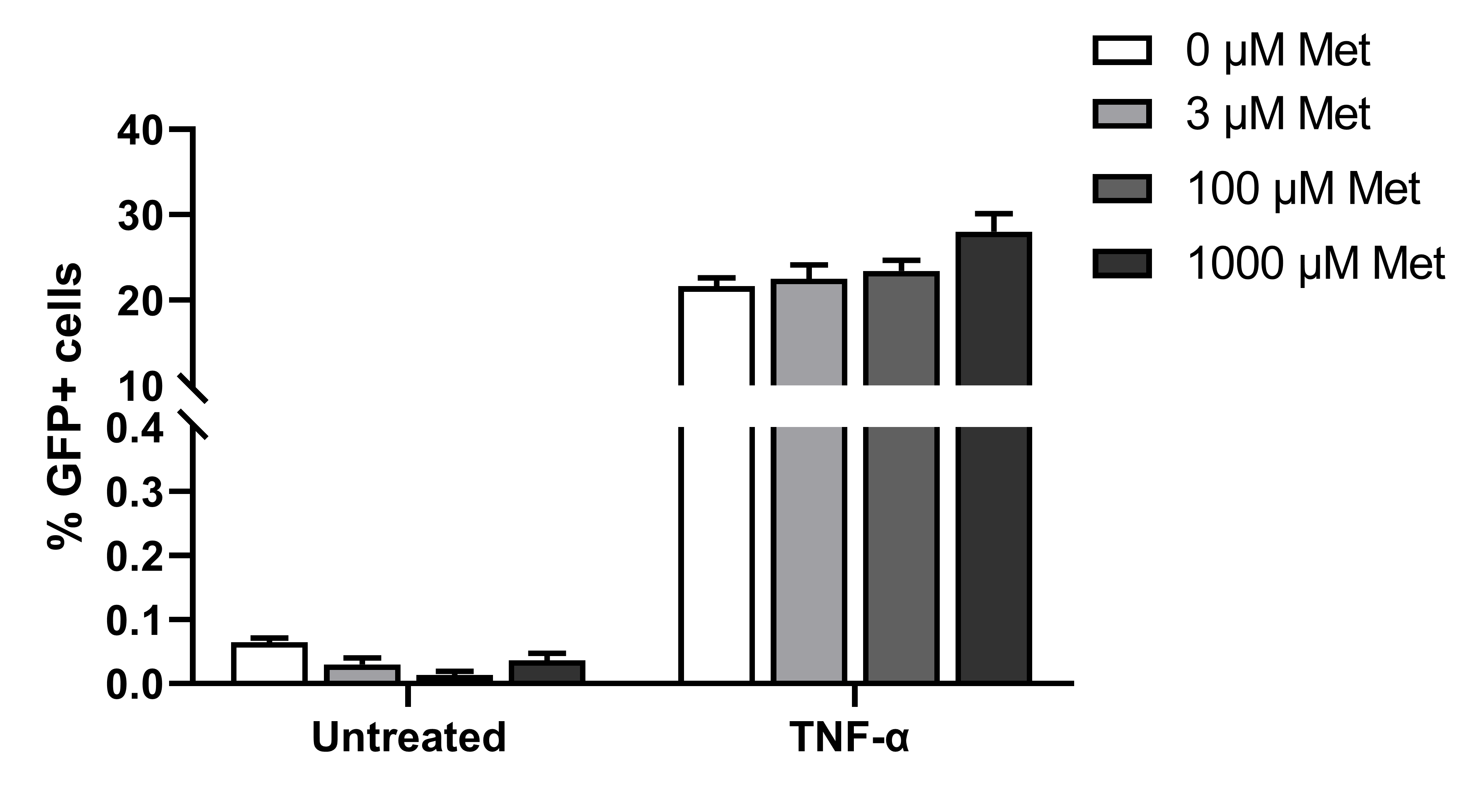

Supplement: Supplementary Figure 1 — MAT1A and MAT2B did not affect the reactivation of latent HIV-1 in J-Lat cells. (A) Schematic diagram of the distribution of MAT1A, MAT2A, and MAT2B in vivo. (B, C) Knockout of MAT1A, MAT2A, and MAT2B in J-Lat 8.4 and J-Lat 9.2 cells, respectively. Flow cytometry analysis of GFP positive cells in J-Lat 8.4 and J-Lat 9.2 cells after TNF-α stimulation. sgNT knockout cells served as control. [file Image_1.tif]

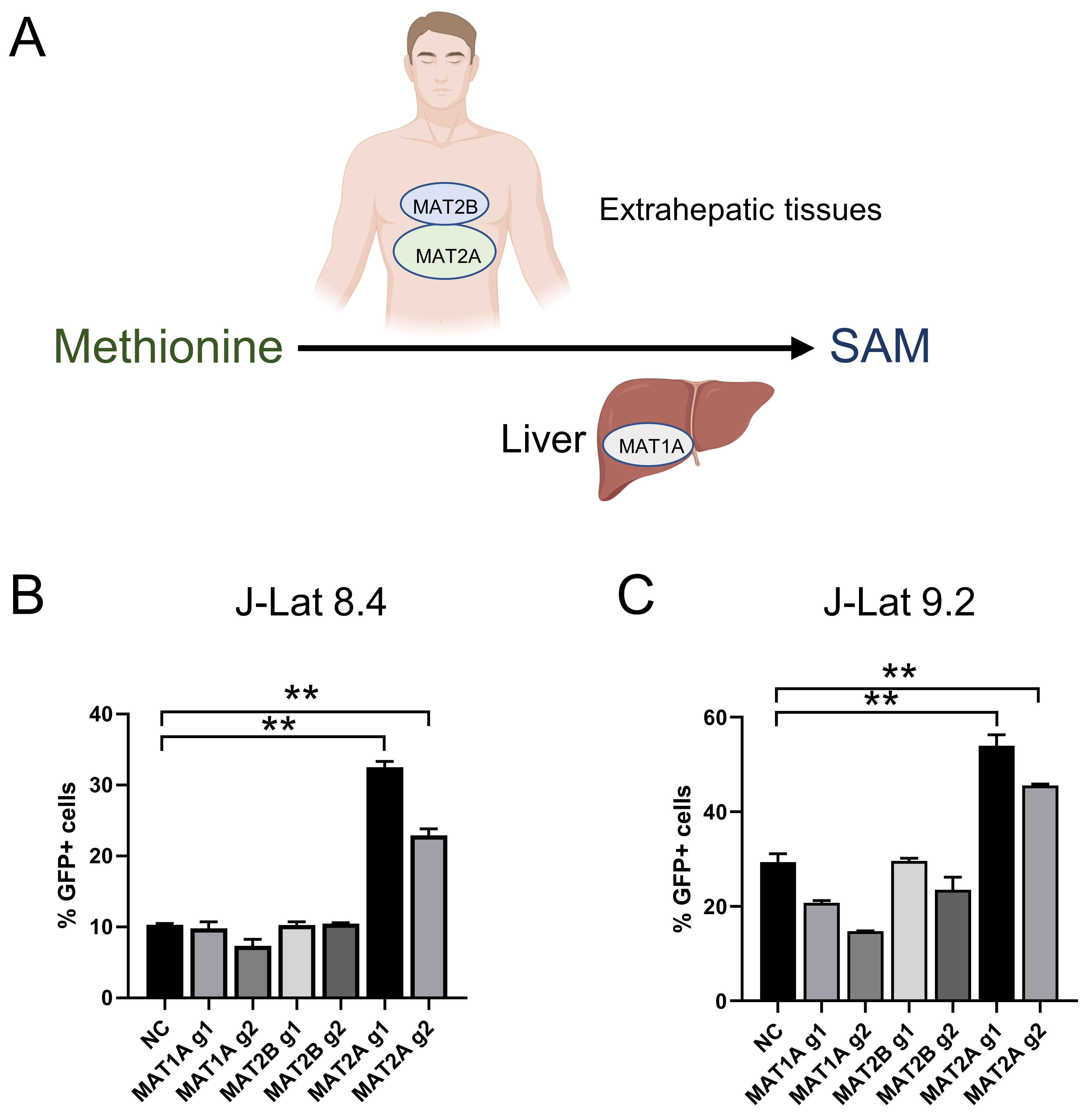

Supplement: Supplementary Figure 2 — Effect of methionine on the reactivation of HIV-1 latency in J-Lat 9.2 cells. J-Lat 9.2 cells were starved with methionine (Met) or supplemented with the indicated concentrations of Met for 7 days and then treated with or without TNF-α for 24 h. GFP-positive cells were measured by flow cytometry. Data were presented as mean ± SD from three independent experiments (Student’s t-test, *p < 0.05, **p < 0.01). [file Image_2.tif]
